# Impact of Preharvest Ethephon Foliar Spray on the Postharvest Fatty Acid Profile and Dietary Indicators of Macadamia Nuts

**DOI:** 10.3390/agriculture13101898

**Published:** 2023-09-27

**Authors:** Noluthando Noxolo Aruwajoye, Asanda Mditshwa, Lembe Samukelo Magwaza, Mjabuliseni Simon Cloapas Ngidi, Samson Zeray Tesfay

**Affiliations:** 1 Discipline of Crop and Horticultural Science, School of Agricultural, Earth and Environmental Sciences, University of KwaZulu-Natal, Private Bag X01, Scottsville, Pietermaritzburg 3209, South Africa; 2 Department of Agricultural Extension and Rural Resource Management, School of Agricultural, Earth and Environmental Sciences, College of Agriculture, Engineering and Science, University of KwaZulu-Natal, Private Bag X01, Scottsville, Pietermaritzburg 3201, South Africa; 3 Centre for Transformative Agricultural and Food Systems, School of Agricultural, Earth and Environmental Sciences, College of Agriculture, Engineering and Science, University of KwaZulu-Natal, Private Bag X01, Scottsville, Pietermaritzburg 3201, South Africa

**Keywords:** ethephon, preharvest, postharvest, ethylene, fatty acids, macadamia, storage

## Abstract

The use of ethephon, designed to stimulate nut detachment, initiates the release of ethylene, a well-established regulator of postharvest shelf-life in various agricultural products. This study aimed to assess the impact of ethephon application on individual fatty acids and dietary indicators in two macadamia nut cultivars, namely ‘788’ and ‘Beaumont,’ during postharvest storage. Nuts that naturally abscised and those detached through ethephon treatment were divided into two groups: the ethylene-treated group (ED) and the control group (CD). Nuts were stored at 25 °C and sampled at 0, 36, and 72 days for fatty profile analysis. Our findings indicated a significant increase in stearic acid content in ED nuts (24,622 µg/g) compared to CD nuts (16,764 µg/g) at the end of the storage period for the ‘Beaumont’ cultivar. Additionally, unsaturated fatty acids (USFAs), such as eicosatrienoic acid + erucic acid (C20:3n^3^ + C22:1) and eicosatrienoic acid + alpha-linolenic acid (C20:1 + C18:3n^3^), were notably reduced. Hierarchical clustering analysis revealed positive correlations between ethylene treatment and saturated fatty acids (SFAs) in both ‘Beaumont’ (0.78) and ‘788’ (0.80) cultivars. This also coincided with an increase in atherogenic indices, thrombogenic index, and saturation index and a decrease in the hypocholesterolemic/hypercholesterolemic ratio and arachidonic acid (C20:4n^6^) within the ED group of the ‘Beaumont’ cultivar, collectively potentially impacting nutritional quality negatively. Furthermore, our findings indicated that the PUFA:SFA ratio was higher in CD (0.51) compared to ED (0.45) on day 72 for the ‘Beaumont’ cultivar, revealing differences in fatty acid compositions between the two treatment groups. Conversely, for ‘788’, both ED and CD had a PUFA:SFA ratio below 0.45, indicating an increased risk of cardiovascular diseases. These results suggest that ethephon treatment increases SFA levels and reduces USFA levels in the ‘Beaumont’ cultivar, while the response to ethylene varies between the two cultivars. Thus, the study provides insight into the significant role of modifying ethephon treatment methods and careful cultivar selection in the attainment of optimal nutritional value and shelf-life of macadamia nuts.

## 1 Introduction

Macadamia nuts, specifically *Macadamia integrifolia* and *Macadamia tetraphylla*, are highly sought after due to their nutritional value and rank among the most economically profitable crop varieties in subtropical regions across the globe [1]. With approximately 75.4 g of fat per 100 g of nuts, macadamia nuts are known for their high fat content [2], which consists of various fatty acids categorized based on the number of double bonds in their carbon chain [3]. These fatty acids fall into three groups: SFAs (saturated fatty acids) if there are no double bonds, MUFAs (monounsaturated fatty acids) if there is one double bond, and PUFAs (polyunsaturated fatty acids) if there is more than one double bond [3]. Notably, monounsaturated fatty acids (MUFAs) form the predominant component of fat in macadamia nuts, comprising up to 54% of the total fat content [4].

**Figure 1:**
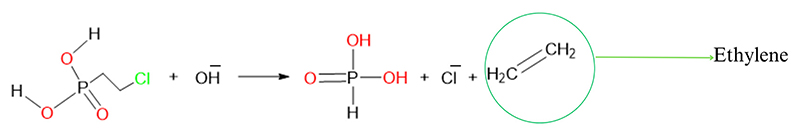
Chemical structure equation depicting the breakdown of ethephon and the release of ethylene.

Ethephon, an ethylene-releasing compound (Figure 1), is commonly used to promote nut abscission during macadamia nut harvesting. This is essential due to the extended nut drop experienced in macadamia, leading to challenges such as multiple harvest periods that incur additional costs and labor-intensive operations [5]. As a natural plant hormone, ethylene plays a crucial role in various physiological processes, which include fruit ripening, senescence, and abscission [6,7]. The fat content of nuts is also correlated with fruit ripening whereby mature crops exhibit a higher ratio of unsaturated fatty acids to saturated fatty acids compared to immature crops [8]. Furthermore, studies have demonstrated the impact of ethylene on fatty acid metabolism. For instance, exogenous ethylene fumigation accelerated the ripening of oil palm fruit and reduced free fatty acid (FFA) levels [9]. Another study reported that ethephon treatment increased the expression of glycerol-3-phosphate acyltransferase (GPAT) in Kentucky bluegrass, resulting in elevated unsaturated fatty acid levels [10]. Moreover, Li [11] found that ethephon treatment led to an upregulation of MdCER6, which is a pivotal gene responsible for the synthesis of ultra-long-chain fatty acids in ‘Starkrimson’ apples [11].

Given ethylene’s significant role in the regulation of postharvest shelf-life for various agricultural products [12], it becomes essential to assess its effect on fatty acids and dietary indicators in macadamia nuts as they serve as a critical quality parameter for both edible nuts and oils [13]. Therefore, this study aims to examine how ethephon treatment affects individual fatty acids and dietary indicators in macadamia nuts that abscised due to the treatment as well as those that naturally abscised during postharvest storage. By investigating the impact of ethephon on fatty acid composition, this research aims to contribute valuable insights into the postharvest quality of macadamia nuts and their suitability for consumption and various applications.

## 2 Materials and Methods

### 2.1 Ethephon Spray and Concentration

**Figure 2:**
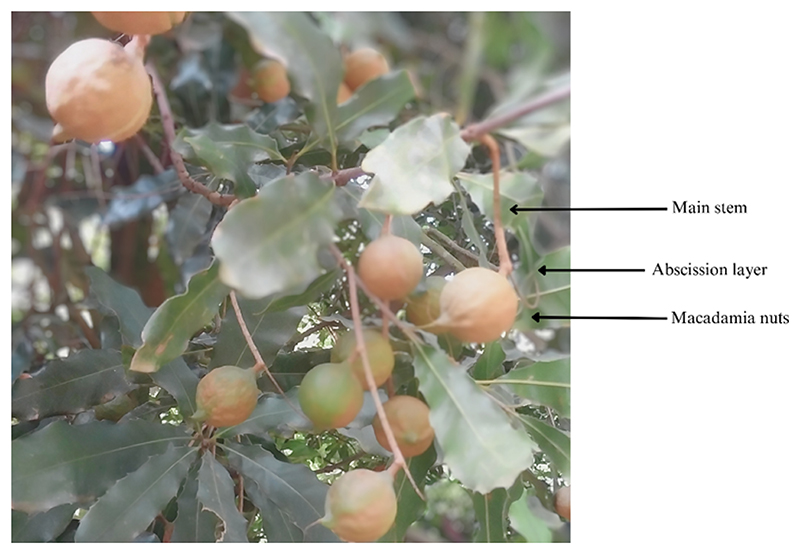
Diagram depicting the role of ethephon in the abscission process of macadamia nuts: stimulated abscission layer for facilitating detachment.

Macadamia nut cultivars ‘Beaumont’ and ‘788’ were harvested from Fyvie estates, uMlaas Road, Camperdown, KwaZulu-Natal, South Africa (latitude: 29°47′50.3′′ S, longitude: 30°27′54′′ E). Line 86–94 ‘The area is characterized by well-drained sandy loam soil, and a range of cultural practices have been implemented to optimize macadamia nut production. These practices encompass drip irrigation for maintaining consistent moisture levels, customized fertilization based on annual soil testing, integrated pest management techniques, regular pruning aimed at enhancing canopy structure and air circulation, careful harvesting at the ideal stage of maturity, and precise application of ethephon to specific macadamia trees to facilitate abscission. Ethylene was administered as ethephon on physiologically mature nuts, serving as a vital plant growth regulator to facilitate nut abscission in macadamia trees [14] (Figure 2). The concentration of ethephon was determined in accordance with industry standards. For ‘788’ macadamia trees, 4 L of ethephon 480 SL per hectare was mixed with 3000 L of water per hectare, resulting in a concentration of 13.33 mL/L. ‘Beaumont’ macadamia trees received a dosage of 5 L of ethephon 480 SL per hectare combined with 3000 L of water per hectare, resulting in a concentration of 16.67 mL/L. Both doses were applied using a Cima mist blower to promote nut abscission. The application of ethephon specifically targeted physiologically mature nuts, and those that naturally detached and fell to the ground were considered successfully abscised nuts.

### 2.2 Nut Collection and Preparation

Freshly harvested nuts, with an initial moisture content of approximately 20.61% on a dry basis (d.b.), included those that had fallen from both ethephon-treated and non-ethephon-treated trees. Nuts with green husks were directly picked from the ground, representing both the ethephon-treated (ED) and control (CD) groups. Following harvesting, the fruits were mechanically dehusked within 24 h, weighed, and subsequently dried to approximately 2.42% on a dry basis (d.b.) using a laboratory Memmert U15 oven from Germany. The drying process involved temperatures starting at 35 °C on the first day, 38 °C on the second day, and 50 °C on the third day, following a modified version of the method outlined by Buthelezi [15].

### 2.3 Storage Conditions

In this research study, a total of 150 macadamia kernels were used for each of the two cultivars (‘788’ and ‘Beaumont’). Within each cultivar, these kernels were divided into two distinct groups: 75 kernels originated from trees subjected to ethephon treatment (ED), representing the treated group, while the remaining 75 kernels were from control trees (CD), which remained untreated. Subsequently, these kernels were organized into three separate replicates each comprising 5 kernels and securely enclosed within conventional polythene bags. These bags, containing the kernels, were then stored at a temperature of 25 °C for a maximum duration of 72 days. The selected storage duration was chosen to simulate accelerated shelf-life studies, which involve elevated storage conditions, frequently with higher temperatures in order to predict how long the product will remain suitable for consumption or use when stored under standard storage conditions [15]. To ensure a comprehensive evaluation of the fatty acid profile, a systematic sampling regimen was implemented, with sampling events conducted at predetermined intervals of 0, 36, and 72 days. These time points were selected to capture any potential dynamics in the fatty acid composition throughout the storage period. After each sampling event, the collected macadamia nuts were preserved at −20 °C, maintaining the integrity and quality of the samples for subsequent in-depth analysis.

### 2.4 Quantification of Fatty Acid Profile

The fatty acid analysis was conducted following modification of the method described by [4,16]. Initially, 100 mg of the sample was weighed, and 2 mL of hexane was added. As an internal standard, 50 µL of 1000 ppm heptadecanoic acid (C17) was also added. The mixture received 1 mL of 20% sulfuric acid in a methanol solution and was then incubated in an oven at 80 °C for one hour. After incubation, the mixture was allowed to cool to room temperature. To extract the fatty acid methyl esters (FAMEs), 3 mL of 20% (*w*/*v*) NaCl was added, and the materials were agitated firmly for phase separation. The upper hexane phase containing FAMEs was transferred to a gas chromatograph (GC) vial. FAMEs were separated using a GC (Trace1300, Thermo Scientific, Austin, TX, USA) connected to a flame ionization detector (FID) on a non-polar Stabil wax column (60 m, 0.32 mm ID, 0.25 m film thickness). Helium at 1 mL/min was employed as the carrier gas, and the injector temperature was maintained at 240 °C. A 5:1 split ratio was used for injecting 1 µL of the material. The oven temperature was programmed as follows: 50 °C for 2 min, increased to 180 °C at a rate of 25 °C per minute and held for 2 min, 200 °C at a rate of 3 °C per minute and held for 5 min, and finally 240 °C at a rate of 4 °C per minute and kept for 15 min. Identification of each FA in the chromatogram was performed by comparing the retention times with certified standard mixes (Grain FAME Mix Supelco, Bellefonte, PA, USA; Catalog No: 47801) using the Chrom-Card data system version 2.3 software for Windows (Thermo Electron, Rodano, Italy). The quantification of FAs was achieved by measuring the peak areas on the chromatogram and expressing the results in grams of FA per gram of total FAs. The obtained fatty acid composition was used to calculate the total fatty acids (TFAs), the ratio of saturated to unsaturated fatty acids (SFA/MUFA), and the ratio of monounsaturated to polyunsaturated fatty acids (MUFA/PUFA).

### 2.5 Dietary Indicators

The dietary indices of macadamia nuts, encompassing the atherogenic index, thrombogenic index, saturation index, lipid nutritional value, and the hypocholesterolemic/hypercholesterolemic ratio, were computed using the equations described by [17], as presented below:

(1) $$AI=\frac{C12:0+4\times C14:0+C16:0}{MUFA+n-6+n-3}$$

(2) $$TI=\frac{C14:0+C16:0+C18:0}{0.5MUFA+0.5n-6+3n-3+\frac{n-3}{n-6}}$$

(3) $$\frac{S}{p}=\frac{C14:0+C16:0+C18:0}{MUFA+PUFA}$$

(4) $$NV=\frac{C12:0+C14:0+C16:0}{C18:1+C18:2}$$

(5) $$\frac{h}{H}=\frac{C16:1+C18:1+C18:2+C20:4n-6}{C12:0+C14:0+C16:0}$$

### 2.6 Data Analysis

Analysis of variance (ANOVA) was performed using GenStat^®^ 20th Edition (VSN International Ltd., Hertfordshire, UK). Means were separated using the least significant difference (LSD) test at 5% levels of significance. Principal component analysis (PCA), biplot diagrams, and hierarchical clustering heat map analysis were utilized in Python programming to comprehensively identify the impact of ethephon on the fatty acid profile of the two macadamia nut cultivars during postharvest storage.

## 3 Results and Discussion

### 3.1 Fatty Acid Profile

Tables 1 and 2 present the results of fatty acid methyl ester (FAME) analysis from ‘Beaumont’ and ‘788’ cultivars after 72 days of storage at 25 °C. The concentration of monounsaturated was the most abundant in both cultivars, particularly oleic acid (C18:1) and palmitoleic acid (C16:1) (see Tables 1 and 2). Macadamia nuts are well known for their abundance of monounsaturated fatty acids (MUFAs) [4,18]. The concentration of oleic acid (C18:1) was not significantly affected by ethephon or storage time. However, there was a significant difference (*p* < 0.001) in their concentrations between the two cultivars, with ‘Beaumont’ exhibiting the highest concentration. Palmitoleic acid (C16:1) was influenced by the interaction effect of Treatment × Day × Cultivar (*p* < 0.001); particularly in the ‘Beaumont’ cultivar, on day 0, ED (156,446 µg/g) exhibited a higher concentration of palmitoleic acid compared to CD (99,582 µg/g), whereas in ‘788’ CD displayed a higher concentration than ED. Macadamia nuts are also known for their low levels of saturated fatty acids (SFAs), with palmitic acid (C16:0) and stearic acid (C18:0) being the most abundant SFAs [18], as indicated in Tables 1 and 2.

In the ‘Beaumont’ cultivar, after 72 days of storage, the concentration of palmitic acid was found to be higher in the ED (62,674 µg/g) harvest compared to the CD (56,628 µg/g) harvest, although this difference was not statistically significant. Nuts from the ED group exhibited a significantly higher stearic acid (C18:0) content at 24,622 µg/g compared to the CD group, which had 16,764 µg/g. In the ‘788’ cultivar, no significant differences in palmitic acid and stearic acid were observed at the end of the storage period. Stearic acid is a long-chain saturated fatty acid known for its association with reductions in low-density lipoprotein cholesterol, high-density lipoprotein cholesterol, and Apolipoprotein A-I levels, as well as an increase in Cholesteryl ester transfer protein mass [19,20]. Moreover, stearic acid is known for its effects on HDL cholesterol levels and holds considerable importance within dietary recommendations due to its potential to influence the crucial ratio of HDL cholesterol to LDL cholesterol, which is a key factor in determining cardiovascular health [21]. These findings are consistent with previous studies by [22], which also reported an increase in saturated fatty acids and a decrease in unsaturated fatty acids in response to ethylene treatment [22]. Another study by [23] also reported that the use of ethylene may reduce unsaturated fatty acids. In this study, we observed a significant decrease in unsaturated fatty acids in ED, particularly Eicosatrienoic acid + Erucic acid (C20:3n^3^ + C22:1) and Eicosatrienoic acid + Alpha-linolenic acid (C20:1 + C18:3n^3^). These changes in fatty acid composition may be due to the effect of ethylene on regulating genes associated with fatty acid biosynthesis [24]. A study by Li [8] found that preharvest ethylene treatment in Camellia oleifera increased the alpha-linolenic acid content by regulating genes involved in linoleic acid and α-linolenic acid metabolism [8]. For eicosatrienoic acid + alpha-linolenic acid (C20:1 + C18:3n^3^), the interaction effect of Treatment × Day × Cultivar on this combination was significant (*p* < 0.05), with CD (8739 µg/g) displaying a higher content compared to ED (6637 µg/g) (Table 1). Moreover, for eicosatrienoic acid + erucic acid (C20:3n^3^ + C22:1), the interaction effect of Treatment × Day × Cultivar on this combination was significant (*p* < 0.05), with CD (938.8) displaying a higher content compared to ED (641 µg/g) (Table 1). In the 788 cultivar, no significant differences were observed in eicosatrienoic acid + erucic acid (C20:3n^3^ + C22:1) between treatments or during storage (Table 2). Unsaturated fatty acids offer various health benefits when incorporated into the diet [25]. However, erucic acid (C22:1) is a monounsaturated omega-9 fatty acid found in numerous plant oils [26]. It is not easily digested and absorbed in the human body, and its potential health risks have led to strict guidelines by regulatory bodies on maximum erucic acid content in oils [27]. Furthermore, at the end of the storage period, the content of arachidonic acid (C20:4n^6^), a type of polyunsaturated fat, was influenced by the interaction of Treatment × Day × Cultivar (*p* < 0.001), with ED (26.38 µg/g) showing a significantly lower content compared to CD (114.8 µg/g) (Table 1). In the 788 cultivar, no significant differences were observed in arachidonic acid (C20:4n^6^) between treatments or during storage (Table 2). Ros [28] reported that MUFAs or n^−6^ PUFAs, such as arachidonic acid (C20:4n^6^), lead to lower blood cholesterol levels and potentially provide beneficial effects on inflammation, thrombosis, and vascular reactivity [28]. Moreover, in the ‘Beaumont’ cultivar, CD (0.51) had a significantly higher PUFA:SFA ratio than ED (0.45) at day 72, indicating distinct fatty acid compositions between the two groups. As discussed earlier, ethephon is known to induce an increase in saturated fatty acids and a decrease in unsaturated fatty acids, which could lead to an overall reduction in the PUFA:SFA ratio. A high PUFA/SFA ratio is recommended due to its health benefits, whereas a PUFA/SFA ratio lower than 0.45 may increase the incidence of cardiovascular diseases [29]. For the ‘Beaumont’ cultivar, all of the nuts had a PUFA/SFA that was equal to or higher than 0.45. A similar trend was observed for the ‘788’, except for on day 72, where both ED and CD were lower than 0.45.

In the ‘Beaumont’ cultivar, the (∑ n^−6^)/(∑ n^−3^) ratio remained consistent for both ED and CD treatments from day 0 to 36. However, by day 72, CD exhibited the lowest ratio (1.59) compared to ED (2.36), suggesting changes in the (∑ n^−6^)/(∑ n^−3^) ratio over the storage period. This decrease in the (∑ n^−6^)/(∑ n^−3^) ratio of CD at day 72 was also observed in cultivar ‘788’. However, the (∑ n^−6^)/(∑ n^−3^) ratio of ED was lower for day 0 and 36 in ‘788’. The ∑ n^−6^)/(∑ n^−3^) represents the ratio of the total omega-6 (n^−6^) polyunsaturated fatty acids (PUFAs) to the total omega-3 (n^−3^) polyunsaturated fatty acids (PUFAs) [30]. A lower ratio of omega-6 to omega-3 PUFAs is believed to have health benefits and may contribute to better overall health and reduced risk of diseases such as nonalcoholic fatty liver disease [31]. A low n^−6^ to n^−3^ polyunsaturated fatty acid (PUFA) ratio of 4:1 was found to result in a significant reduction of approximately 30% in hepatic fat content among obese youth with nonalcoholic fatty liver disease [32]. For both cultivars, the (∑ n^−6^)/(∑ n^−3^) ratio was found to be within the acceptable low ratio of 4:1 or lower.

### 3.2 Principal Component Analysis (PCA)

**Figure 3:**
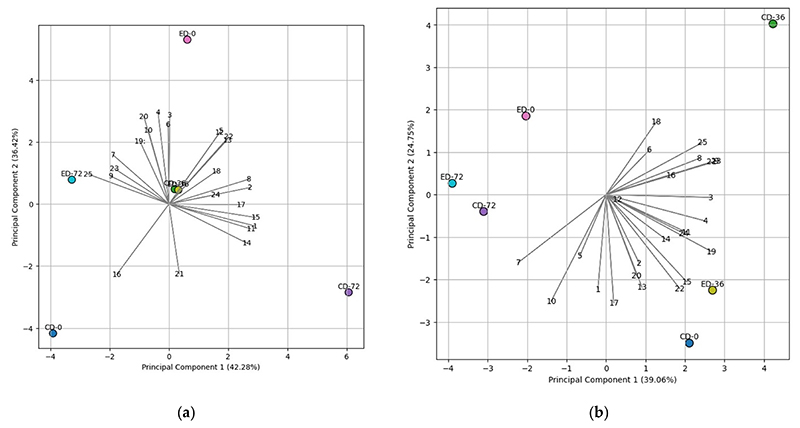
The biplot of Principal Component 1 (PC1) versus Principal Component 2 (PC2) illustrates the relationship between fatty acids and different treatments for (**a**) ‘Beaumont’ and (**b**) ‘788’ cultivars during storage. The treatments are denoted as CD for control drop and ED for ethephon drop, and the storage days are represented as 0, 36, and 72. Specific numerical representations were assigned to various fatty acid components for clarity and ease of reference. These numerical assignments are as follows: 1 represents C12, 2 represents C14, 3 represents C15, 4 represents C16, 5 represents C16:1, 6 represents C17, 7 represents C18, 8 represents C18:1 (cis), 9 represents C18:2 (cis), 10 represents C20, 11 represents C20:1 + C18:3n^3^, 12 represents C21, 13 represents C22, 14 represents C20:3n^3^ + C22:1, 15 represents C20:4n^6^, 16 represents C23, 17 represents C24, 18 represents PUFA (polyunsaturated fatty acid), 19 represents SFA (saturated fatty acid), 20 represents PUFA:SFA, 21 represents TFA = ∑ (Sum of all fatty acids), 22 represents Omega-6 (n^−6^), 23 represents Omega-3 (n^−3^), and 24 represents ∑ n^−6^)/(∑ n^−3^).

PCA has been used as a statistical analysis method for data reduction in various data analyses [33]. In this study, it was employed to identify the most relevant fatty acid variables and their correlations with specific treatments, providing insights into the impact of treatments on the fatty acid composition of the nuts. For the ‘788’ cultivar, PC1 accounted for 39.06%, and PC2 accounted for 24.75% of the total variability (Figure 3b). In the PCA analysis of the ‘788’ cultivar, the influence of the ethephon treatment was observed in the ED-36 group, where it showed a close association with PUFAs (Figure 3b). For the ‘Beaumont’ cultivar, PC1 accounted for 43.28%, and PC2 accounted for 36.42% of the total variability (Figure 3a). The (∑ n^−6^)/(∑ n^−3^) ratio and stearic acid (C18:0) exhibited a positive correlation with the ED-72 treatment, as indicated in Figure 3a. On the other hand, eicosatrienoic acid + erucic acid (C20:3n^3^ + C22:1), arachidonic acid (C20:4n^6^), lauric acid (C12:0), and PUFA:SFA ratio vectors pointed toward the CD-72 treatment, implying a positive correlation, as shown in Figure 3a. These results are also consistent with the findings presented in Tables 1 and 2.

### 3.3 Hierarchical Clustering Heat Map and Correlation Analysis

**Figure 4:**
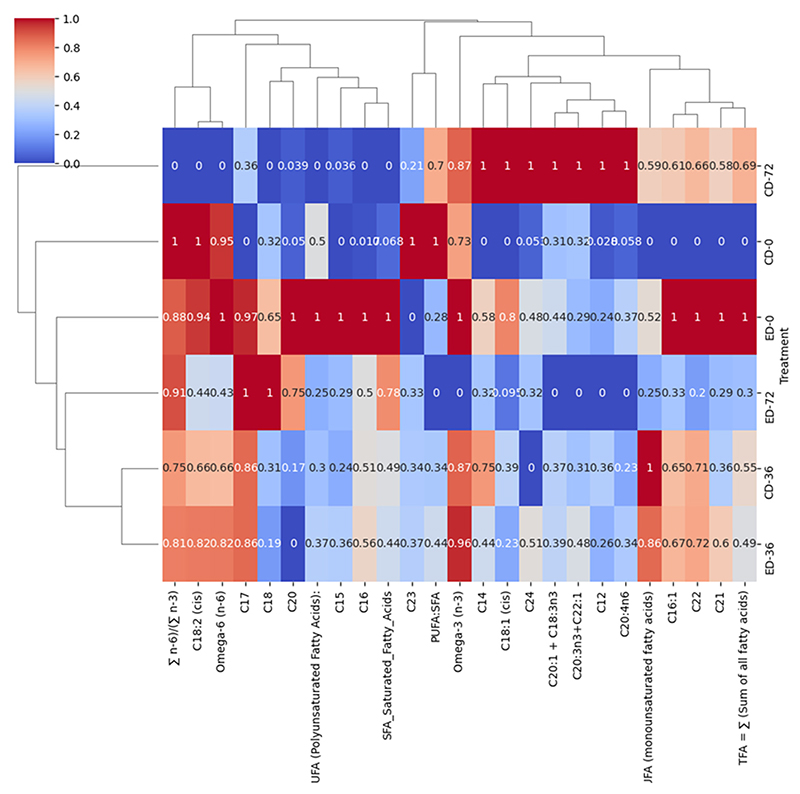
Hierarchical clustering heat map of fatty acids in the ‘Beaumont’ cultivar during postharvest storage. The treatments are denoted as CD for control drop and ED for ethephon drop, and the storage days are represented as 0, 36, and 72.

In the ‘Beaumont’ cultivar, at the end of the storage period, there was a significant positive correlation (0.78) between SFAs and ED-72, while a negative correlation (0.25) was observed between SFAs and PUFAs. As a result, there was a negative correlation between ED-72 and the PUFA:SFA ratio. This negative correlation indicates that the application of ethylene treatment resulted in a reduction in the ratio of polyunsaturated fatty acids to saturated fatty acids. This decrease can be attributed to the increase in SFA levels, which, in turn, led to a decrease in the overall proportion of polyunsaturated fatty acids. Polyunsaturated fatty acids, namely eicosatrienoic acid + alpha-linolenic, eicosatrienoic acid + erucic acid, and arachidonic acid, were strongly correlated (1) with CD-72. Oleic acid, one of the most abundant MUFAs in macadamia nuts [4,18], was also strongly correlated (1) with CD-72, while SFAs, namely myristic acid, lauric acid, and tetracosanoic acid, were strongly correlated (1) with CD-72 and stearic acid (18:0), heptadecanoic acid (C17:0), and arachidic acid (C20:0) exhibited a strong positive correlation of 1 with ED-72 (Figure 4). Moreover, the (∑ n^−6^)/(∑ n^−3^) was strongly correlated (0.91) with ED-72; this is similar to the observation from PCA analysis.

**Figure 5:**
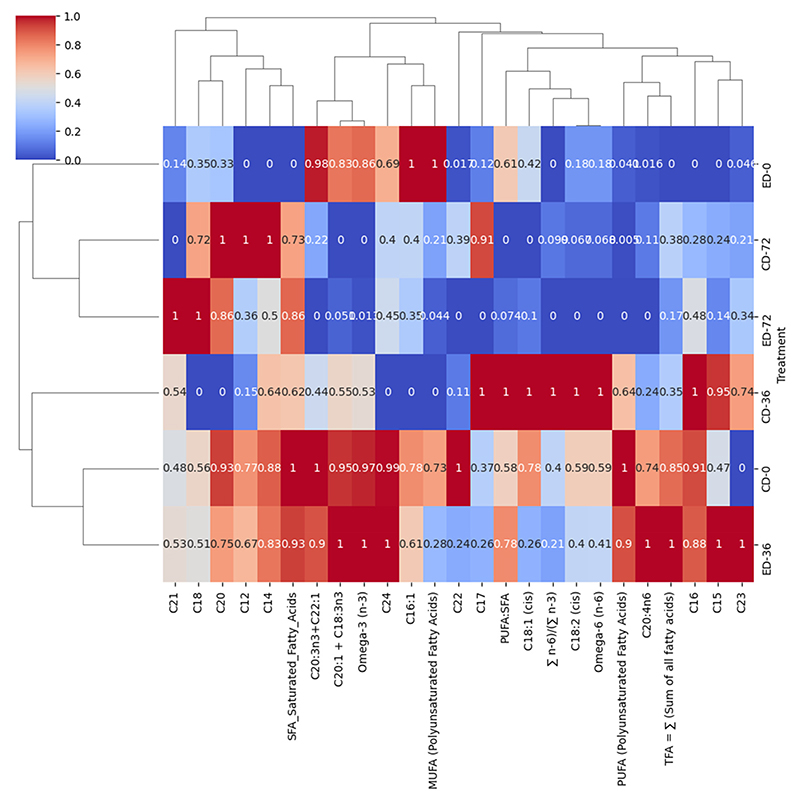
Hierarchical clustering heat map of fatty acids in the ‘788’ cultivar during postharvest storage. The treatments are denoted as CD for control drop and ED for ethephon drop, and the storage days are represented as 0, 36, and 72.

In the ‘788’ cultivar (Figure 5), at the outset (CD-0), there was a strong positive correlation (correlation coefficient of 1) between total SFAs and the control group (CD), suggesting that the SFA content is high in the control group right after harvesting. In contrast, the correlation with the ethephon-treated group (ED) was negative (correlation coefficient of 0), indicating that the SFA content was initially lower in the ED group. As time progressed to day 36, the correlation between total SFA and both ED and CD increased. For ED, the correlation rose significantly to 0.93, indicating that the SFA content in the ethephon-treated group becomes more aligned with the control group. Similarly, for CD, the correlation also increased to 0.64, suggesting that the SFA content in the control group undergoes some changes. Finally, at the end of the storage period, the correlation remained high for both ED and CD, with ED having a correlation coefficient of 0.86 and CD having a correlation coefficient of 0.73. This suggests that, over time, the SFA content in both groups converges. The rise in the total SFA content in CD-72 could potentially be explained by the fact that lauric acid (with a correlation coefficient of 1), myristic acid (also with a correlation coefficient of 1), heptadecanoic acid (with a correlation coefficient of 0.91), and arachidic acid (again with a correlation coefficient of 1) exhibited strong positive correlations with CD-72. Furthermore, the increase in SFA content in ED-72 can be attributed to the positive correlations observed with specific saturated fatty acids, including stearic acid (18:0) (correlation coefficient of 1), arachidic acid (C20:0) (correlation coefficient of 0.86), and heneicosanoic acid (C21:0) (correlation coefficient of 1). These SFAs exhibited a strong positive association with ED-72.

At day 0, there was a negative correlation of 0.40 between PUFAs and ED, indicating that the initial PUFA content is lower in the ethephon-treated group compared to the control group (CD). This suggests that ethephon treatment may have initially affected the PUFA levels in the macadamia nuts. In contrast, PUFAs were positively correlated (correlation coefficient of 1) with CD, implying that the control group had a higher PUFA content at the beginning of the study.

As the storage period progressed to day 36, PUFAs became strongly correlated with both ED (correlation coefficient of 0.90) and CD (correlation coefficient of 0.64). This suggests that over time, the PUFA content in both groups becomes more similar, possibly due to changes in storage conditions or natural ripening processes. At day 72, PUFAs showed no correlation (correlation coefficient of 0) with either CD or ED. Regarding the PUFA:SFA ratio, at day 0, it had a correlation of 0.61 with ED and 0.58 with CD. This suggests that the initial PUFA:SFA ratios are similar between the two groups. However, by day 36, the PUFA:SFA ratio was strongly correlated with ED (correlation coefficient of 0.78) and highly correlated with CD (correlation coefficient of 1). This indicates that both groups see an increase in PUFA:SFA ratios over the storage period, with CD showing a particularly pronounced change. At day 72, the PUFA:SFA ratio was negatively correlated with both CD and ED. However, it is worth noting that the impact of ethylene treatment on fatty acid composition varied between the ‘Beaumont’ and ‘788’ cultivars. This indicates that the response to ethylene treatment might differ depending on the specific cultivar and the stage of storage [34].

### 3.4 Dietary Indices

Table 3 illustrates the impact of ethephon on the dietary indices of the ‘Beaumont’ and ‘788’ cultivars during postharvest storage. The atherogenic index (AI), thrombogenic index (TI), and hypocholesterolemic/hypercholesterolemic ratio (h/H) are employed as predictors of cardiovascular risk and improved nutritional value. Lower AI and TI values, along with a high h/H ratio, are considered preferable for this purpose [35]. In the case of the ‘Beaumont’ cultivar, the application of ED led to an increase in atherogenic indices, the thrombogenic index, the saturation index, and lipid nutritional value. However, it also resulted in a reduction in the hypocholesterolemic/hypercholesterolemic ratio to 7.16, as opposed to the CD group, which exhibited a ratio of 8.61 on day 72. Elevated atherogenic index (AI) and thrombogenic index (TI) values are implicated in atheroma development and the promotion of platelet aggregation within the cardiovascular system [36]. The thrombogenicity index signifies a predisposition to blood clot formation and has been closely linked to stearic acid [37]. Stearic acid is generally believed to have a neutral impact in terms of atherogenicity. However, it is now being considered as having thrombogenic properties instead [38–41]. Therefore, the increase in the thrombogenic index might arise from the significant elevation in stearic acid observed in the ‘Beaumont’ cultivar under ED conditions as well as the strong correlation observed between ED and SFAs. Maintaining lower stearic acid levels holds advantages in preventing cardiovascular disorders. Consequently, the application of ethephon to the ‘Beaumont’ cultivar might yield unfavorable outcomes. Hence, prioritizing lower values is deemed desirable. Regarding the ‘788’ cultivar, at the culmination of the storage period, notable differences in atherogenic indices or the thrombogenic index between the CD and ED groups were not observed. Nevertheless, there was an elevated hypocholesterolemic/hypercholesterolemic ratio in the ED group, indicating a distinct cultivar-specific response to ethephon.

The aforementioned results hold implications for nutrition security, a facet aimed at tackling hidden hunger as part of Sustainable Development Goal (SDG) number 2: zero hunger. Nuts play a pivotal role in ensuring nutrition security and are generally favored by the public for their nutritional benefits. However, the observed increase in stearic acid levels, atherogenic indices, and the thrombogenic index, coupled with a reduction in the hypocholesterolemic/hypercholesterolemic ratio within the ‘Beaumont’ cultivar treated with ethephon, indeed presents a detrimental impact on its nutritional value.

## 4 Conclusions

The present study provides valuable insight into the influence of ethephon on the fatty acid composition and dietary indicators of ‘Beaumont’ and ‘788’ macadamia nut cultivars that abscised due to treatment and without treatment during their postharvest storage period. Our findings reveal significant changes in some fatty acid profiles, particularly in ‘Beaumont,’ where an increase in saturated fatty acids (SFAs) and a decrease in unsaturated fatty acids were observed. This could adversely impact the nutritional quality of the nuts. However, to comprehensively evaluate the effect of ethephon treatment in light of the nutritional implications, future studies should incorporate sensory evaluations. Additionally, for the ‘Beaumont’ cultivar, ethephon application resulted in an increase in atherogenic and thrombogenic indices, the saturation index, and lipid nutritional value while concurrently decreasing the hypocholesterolemic/hypercholesterolemic ratio. These changes, particularly the elevated thrombogenic index, could potentially pose cardiovascular risks. On the other hand, the ‘788’ cultivar produced no significant differences in atherogenic or thrombogenic indices but an increased hypocholesterolemic/hypercholesterolemic ratio, indicating a cultivar-specific effect. Thus, this study hints at the potential for refining strategies to optimize the nutritional value and shelf-life of macadamia nuts, which can be achieved by modifying ethephon treatment methods and carefully selecting cultivars. Specifically, by adjusting ethephon concentrations, application timings, and methodologies, producers can minimize adverse effects on fatty acid composition while maintaining desired ripening effects in macadamia nuts. Additionally, the selection of macadamia cultivars less sensitive to changes in fatty acid profiles can help preserve a more desirable composition in the final product.

## Figures and Tables

**Table 1:** The fatty acid compositions (µg/g) of ‘Beaumont’ macadamia kernels from the ethylene-treated group (ED) and the control group (CD) during postharvest storage.

Treatment	Day	Lauric Acid (C12:0)	Myristic Acid (C14:0)	Pentadecanoic Acid (C15:0)	Palmitic Acid (C16:0)	Palmitoleic Acid (C16:1)	Heptadecanoic Acid (C17:0)	Stearic Acid (C18:0)
CD	0	395.1 ± 18.08 ab	3755 ± 130.79 abc	70.02 ± 4.10 ab	56,838 ± 2561.39 abc	99,582 ± 4990.18 ab	162.9 ± 11.19 a	19,291 ± 658.01 abc
ED	0	437.6 ± 16.25 ab	4566 ± 317.24 bcd	90.53 ± 9.11b	68,632 ± 4236.48 c	156,446 ± 9299.51 e	219.8 ± 8.08 a	21,872 ± 1652.29 bc
CD	36	462.3 ± 27.09 ab	4815 ± 107.35 cd	75.04 ± 10.54 ab	62,736 ± 1156.96 bc	136,538 ± 3511.92 de	213.4 ± 16.08 a	19,178 ± 107.52 abc
ED	36	442.4 ± 5.05 ab	4382 ± 9.46 bcd	77.38 ± 2.38 ab	63,297 ± 669.35 bc	137,833 ± 1508.76 de	213.4 ± 0,91 a	18,265 ± 164.54 ab
CD	72	592.5 ± 12.29 c	5165 ± 51.19 d	70.76 ± 1.83 ab	56,628 ± 1306.99 abc	134,027 ± 2922.03 d	184.0 ± 8.86 a	16,764 ± 170.26 ab
ED	72	389.4 ± 4.85 ab	4210 ± 83.21 bcd	75.91 ± 2.62 ab	62,674 ± 1034.34 bc	118,159 ± 2807.36 bcd	221.7 ± 37.56 a	24,622 ± 658.74 c
Treatment	Day	Oleic Acid C18:1 (cis)	Linoleic Acid C18:2 (cis)	Arachidic Acid (C20:0)	Eicosatrienoic Acid + Alpha-linolenic Acid (C20:1 + C18:3n^3^)	Heneicosanoic Acid (C21:0)	Docosanoic Acid (C22:0)	Eicosatrienoic Acid + Erucic Acid (C20:3n^3^ + C22:1)
CD	0	342,102 ± 10,793.36 abc	19,530 ± 798.08 f	15,283 ± 587.35 ab	7279 ± 209.79 bcd	35.44 ± 6.34 ab	4176 ± 101.99 abc	737.4 ± 15.42 cd
ED	0	378,430 ± 25,126.64 bc	19,290 ± 1146.71 ef	17,771 ± 1324.36 b	7559 ± 492.45 cd	55.98 ± 7.79 b	5137 ± 203.35 c	726.5 ± 45.03 cd
CD	36	359,739 ± 5505.98 abc	18,095 ± 439.15 ef	15,595 ± 365.73 ab	7422 ± 160.33 cd	42.76 ± 2.39 ab	4858 ± 240.69 bc	732.9 ± 17.59 cd
ED	36	352,704 ± 2704.59 abc	18,764 ± 210.28 ef	15,151 ± 113.74 ab	7465 ± 100.44 cd	47.85 ± 5.16 ab	4865 ± 167.58 bc	783.2 ± 13.18 de
CD	72	387,473 ± 4298.73 c	15,316 ± 471.14 cde	15,252 ± 119.84 ab	8739 ± 84.61 d	47.37 ± 2.85 ab	4811 ± 177.05 abc	938.8 ± 10.98 e
ED	72	346,413 ± 4826.80 abc	17,173 ± 442.70 def	17,128 ± 378.03 b	6637 ± 117.11 abc	41.35 ± 2.79 ab	4366 ± 189.99 abc	641 ± 13.15 bcd
Treatment	Day	Arachidonic Acid (C20:4n^6^)	Tricosanoic Acid (C23:0)	Tetracosanoic Acid (C24:0)	MUFA	PUFA	SFA	PUFA:SFA
CD	0	31.53 ± 4.42 a	68.46 ± 3.68 a	1924 ± 45.91 bcde	441,684 ± 15,772.48 abc	42,860 ± 1585.13 de	80,512 ± 3342.11 abcd	0.53 ± 0.01 g
ED	0	59.27 ± 14.13 ab	48.25 ± 6.97 a	2020 ± 129.79 de	534,876 ± 34,417.53 c	45,406 ± 2994.74 e	95,818 ± 6208.41 d	0.47 ± 0 de
CD	36	46.68 ± 6.27 a	55.12 ± 1.71 a	1912 ± 43.31 bcde	496,278 ± 8416.62 abc	41,891 ± 958.29 cde	87,480 ± 1366.18 bcd	0.48 ± 0 e
ED	36	56.5 ± 2.71 a	55.66 ± 0.70 a	2027 ± 29.26 de	490,537 ± 4113.63 abc	42,219 ± 424.29 cde	86,677 ± 846.50 bcd	0.49 ± 0 ef
CD	72	114.8 ± 26.09 b	52.59 ± 2.89 a	2137 ± 29.270 e	521,500 ± 5037.15 bc	40,360 ± 619.07 bcde	79,404 ± 1529.48 abcd	0.51 ± 0 fg
ED	72	26.38 ± 4.56 a	54.88 ± 1.69 a	1983 ± 47.90 cde	464,572 ± 7351.12 abc	41,605 ± 931.15 cde	92,193 ± 1768.74 cd	0.45 ± 0bcd
Treatment	Day	TFA	Omega-6 (n^−6^)	Omega-3 (n^-3^)	∑ n^−6^)/(∑ n^−3^)			
CD	0	571,240 ± 12,614.22 abc	19,350 ± 797.97 e	8016 ± 225.19 bc	2.44 ± 0.03 e			
ED	0	683,381 ± 450,303.65 c	19,561 ± 1160.79 e	8285 ± 537.43 bc	2.31 ± 0.03 de			
CD	36	632,517 ± 20,433.35 abc	18,141 ± 439.6 e	8155 ± 177.72 bc	2.21 ± 0.02 d			
ED	36	626,429 ± 4658.12 abc	18,820 ± 211.31 e	8249 ± 113.50 bc	2.28 ± 0.02 de			
CD	72	648,314 ± 13,223.65 bc	15,431 ± 474.97 cde	8155 ± 94.61 bc	1.59 ± 0.05 c			
ED	72	604,813 ± 13,609.36 abc	17,199 ± 444.30 de	7278 ± 130.23 ab	2.36 ± 0.04 de			

CD (control drop), ED (ethephon drop) ∑ (sum), SFA (saturated fatty acid), MUFA (monounsaturated fatty acid), PUFA (polyunsaturated fatty acid), PUFA:SFA (polyunsaturated fatty acid: saturated fatty acid), TFA (total fatty acid). Values are the mean ± SE. Lowercase letters (a–g) indicate differences between treatments during storage.

**Table 2:** The fatty acid compositions (µg/g) of ‘788’ macadamia kernels from the ethylene-treated group (ED) and the control group (CD) during postharvest storage.

Treatment	Day	Lauric Acid (C12:0)	Myristic Acid (C14:0)	Pentadecanoic Acid (C15:0)	Palmitic Acid (C16:00)	Palmitoleic Acid (C16:1)	Heptadecanoic Acid (C17:0)	Stearic Acid (C18:0)
CD	0	456.1 ± 52.38 ab	3856 ± 560.64 abc	58.21 ± 5,83 a	49,028 ± 6394.12 ab	116,149 ± 15,516.71 bcd	241.3 ± 39.24 a	18,472 ± 2361.10 ab
ED	0	340.1 ± 20.96 a	2704 ± 140.84 a	49.53 ± 1.21 a	41,870 ± 2049.04 a	93,446 ± 5644.47 a	219.2 ± 8.93 a	16,904 ± 1051.49 ab
CD	36	362.4 ± 14.82 a	3540 ± 160.33 ab	67.17 ± 5.16 ab	49,733 ± 1705.95 ab	105,114 ± 2412.54 abc	297.0 ± 40.63 a	14,310 ± 617.25 a
ED	36	441.5 ± 28.44 ab	3793 ± 212.98 abc	68.13 ± 6.77 ab	48,819 ± 2740.12 ab	122,610 ± 5727.06 cd	231.7 ± 17.53 a	18,092 ± 865.77 ab
CD	72	491.3 ± 9.31 bc	4018 ± 134.20 bcd	54 ± 1.71 a	44,103 ± 1424.11 a	111,163 ± 4861.47 abcd	289.5 ± 60.94 a	19,711 ± 124.08 abc
ED	72	394.6 ± 6.71 ab	3362 ± 98.02 ab	52.14 ± 1.14 a	45,678 ± 1431.26 a	103,515 ± 2746.10 abc	208.8 ± 9.35 a	21,783 ± 766.09 bc
Treatment	Day	Oleic Acid C18:1 (cis)	Linoleic Acid C18:2 (cis)	Arachidic Acid (C20:0)	Eicosatrienoic Acid + Alpha-linolenic Acid (C20:1 + C18:3n^3^)	Heneicosanoic Acid (C21:00)	Docosanoic Acid (C22:00)	Eicosatrienoic Acid + Erucic Acid (C20:3n^3^ + C22:1)
CD	0	297,263 ± 41,541.23 abc	11,257 ± 1723.93 abc	14,227 ± 1899.80 ab	6191 ± 888.11 abc	27.53 ± 6.58 ab	4146 ± 566.59 abc	598.8 ± 90.53 abcd
ED	0	290,300 ± 8623.62 ab	8630 ± 315.15 a	12,548 ± 416.73 a	6071 ± 162.57 abc	22.69 ± 0.47 a	3581 ± 133.90 a	595.5 ± 21.31 abcd
CD	36	301,545 ± 10,891.06 abc	13,898 ± 585.06 bcd	11,641 ± 405.32 a	5800 ± 231.48 abc	28.36 ± 4.54 ab	3632 ± 129.58 ab	503.8 ± 12.77 ab
ED	36	287,033 ± 18,356.90 ab	10,086 ± 441 ab	13,709 ± 744.47 ab	6241 ± 392.35 abc	28.14 ± 7.28 ab	3707 ± 268.38 ab	582.3 ± 37.33 abc
CD	72	282,001 ± 8719.03 a	7938 ± 375.11 a	14,415 ± 182.85 ab	5250 ± 156.46 a	20.79 ± 3.05 a	3795 ± 58.76 ab	468.3 ± 15.66 ab
ED	72	284,023 ± 9958.49 a	7508 ± 220.39 a	14,021 ± 570.28 ab	5301 ± 218.14 ab	34.69 ± 9.14 ab	3571 ± 163.07 a	430.6 ± 29.59 a
Treatment	Day	Arachidonic Acid (C20:4n^-6^)	Tricosanoic Acid (C23:0)	Tetracosanoic Acid (C24:0)	MUFA	PUFA	SFA	PUFA:SFA
CD	0	23.1 ± 9.94 a	32.99 ± 5.69 a	1573 ± 225.10 abcd	413,412 ± 57,056.50 abc	32,297 ± 4592.10 abcd	72,112 ± 9394.56 abc	0.45 ± 0.01 bc
ED	0	8.29 ± 42.10 a	33.79 ± 0.52 a	1518 ± 48.96 abcd	383,745 ± 14,244.28 a	27,853 ± 875.29 a	62,086 ± 3233.89 a	0.45 ± 0.01 bcd
CD	36	12.77 ± 4.57 a	45.92 ± 19.07 a	1392 ± 58.131 a	392,147 ± 13,243.63 ab	30,647 ± 1180.94 ab	68,309 ± 249.77 ab	0.49 ± 0 ef
ED	36	28.33 ± 14.98 a	50.43 ± 3.31 a	1574 ± 109.31 abcd	424,156 ± 24,051.43 abc	31,855 ± 1615.99 abc	71,446 ± 3864.65 abc	0.47 ± 0 cde
CD	72	10.18 ± 3.20 a	36.7 ± 1.51 a	1464 ± 38.19 ab	395,186 ± 13,574 ab	27,686 ± 625.17 a	69,407 ± 1482.83 ab	0.39 ± 0 a
ED	72	7.97 ± 4.73 a	38.89 ± 4.03 a	1473 ± 59.37 abc	385,517 ± 12,657.02 a	27,663 ± 1012.57 a	70,739 ± 2287.67 abc	0.40 ± 0 a
Treatment	Day	TFA	Omega-6 (n ^-6^)	Omega-3 (n^-3^)	∑ n^−6^)/(∑ n^−3^)			
CD	0	523,601 ± 97,815 abc	11,280 ± 1733.68 abc	6790 ± 978.47 ab	1.66 ± 0.003 c			
ED	0	478,839 ± 28,603.60 a	8638 ± 313.04 a	6667 ± 183.87 ab	1.3 ± 0.03 a			
CD	36	497,414 ± 31,833.44 ab	13,910 ± 580.49 bcd	6304 ± 235.52 ab	2.21 ± 0.04 d			
ED	36	531,603 ± 28,788.47 abc	10,115 ± 455.94 ab	6824 ± 429.62 ab	1.49 ± 0.03 bc			
CD	72	498,714 ± 11,740.23 ab	7948 ± 378.29 a	5718 ± 172.10 a	1.39 ± 0.02 ab			
ED	72	48,7919 ± 24,421 ab	7516 ± 222.80 a	5732 ± 233.23 a	1.30 ± 0.02 a			

CD (control drop), ED (ethephon drop) ∑ (sum), SFA (saturated fatty acid), MUFA (monounsaturated fatty acid), PUFA (polyunsaturated fatty acid), PUFA:SFA (polyunsaturated fatty acid: saturated fatty acid), TFA (total fatty acid). Values are the mean ± SE. Lowercase letters (a–f) indicate differences between treatments during storage.

**Table 3:** Highlights dietary indices of two macadamia cultivars (‘Beaumont’ and ‘788’) throughout postharvest storage.

Beaumont	Treatment	Day	Atherogenic Indices	Thrombogenic Index	Saturation Index	Lipid Nutritional Value	Hypocholesterolemic/Hypercholesterolemic Ratio
	CD	0	0.1539 ± 0 ef	0.3346 ± 0 ef	0.1648 ± 0 fgh	0.1685 ± 0bc	7.569 ± 0.08 b
	ED	0	0.1553 ± 0fg	0.3331 ± 0 ef	0.1638 ± 0 efgh	0.1852 ± 0 f	7.525 ± 0.02 b
	CD	36	0.1578 ± 0 g	0.3268 ± 0 ed	0.1612 ± 0def	0.1800 ± 0 ef	7.564 ± 0.01 b
	ED	36	0.1570 ± 0fg	0.3268 ± 0 ed	0.1613 ± 0 defg	0.1834 ± 0 ef	7.477 ± 0.01 b
	CD	72	0.1425 ± 0 b	0.2824 ± 0 a	0.1398 ± 0 a	0.1549 ± 0.01 a	8.61 ± 0.13 e
	ED	72	0.1634 ± 0h	0.3687 ± 0 g	0.1808 ± 0i	0.1850 ± 0 f	7.162 ± 0.04 a
**788**	**Treatment**	**Day**	**Atherogenic Indices**	**Thrombogenic Index**	**Saturation Index**	**Lipid Nutritional Value**	**Hypocholesterolemic/Hypercholesterolemic Ratio**
	CD	0	0.1506 ± 0 de	0.3263 ± 0.01 de	0.1604 ± 0 de	0.1733 ± 0 cd	7.948 ± 0.06 c
	ED	0	0.1328 ± 0 a	0.3027 ± 0.01 b	0.1492 ± 0 b	0.1501 ± 0.01 a	8.748 ± 0.12 e
	CD	36	0.1558 ± 0fg	0.3228 ± 0 cd	0.1594 ± 0 d	0.1783 ± 0 de	7.571 ± 0.01 b
	ED	36	0.1461 ± 0 c	0.3158 ± 0 c	0.1555 ± 0 c	0.1703 ± 0bc	8.185 ± 0.03 d
	CD	72	0.1484 ± 0 cd	0.3375 ± 0.01 f	0.1654 ± 0 gh	0.1665 ± 0 b	8.292 ± 0.06 d
	ED	72	0.1493 ± 0 cd	0.3399 ± f	0.1664 ± 0h	0.1501 ± 0 a	8.748 ± 0.02 e

CD (control drop) and ED (ethephon drop). Values are the mean ± SE. Lowercase letters (a–i) indicate differences between treatments during storage.

## Data Availability

Data are contained within the article.
